# Application of radiomics and machine learning in head and neck cancers

**DOI:** 10.7150/ijbs.55716

**Published:** 2021-01-01

**Authors:** Zhouying Peng, Yumin Wang, Yaxuan Wang, Sijie Jiang, Ruohao Fan, Hua Zhang, Weihong Jiang

**Affiliations:** Department of Otolaryngology Head and Neck Surgery, Xiangya Hospital, Central South University, Changsha 410078, Hunan, China.

**Keywords:** radiomics, machine learning, head and neck cancers, sequential treatment, big data

## Abstract

With the continuous development of medical image informatics technology, more and more high-throughput quantitative data could be extracted from digital medical images, which has resulted in a new kind of omics-Radiomics. In recent years, in addition to genomics, proteomics and metabolomics, radiomic has attracted the interest of more and more researchers. Compared to other omics, radiomics can be perfectly integrated with clinical data, even with the pathology and molecular biomarker, so that the study can be closer to the clinical reality and more revealing of the tumor development. Mass data will also be generated in this process. Machine learning, due to its own characteristics, has a unique advantage in processing massive radiomic data. By analyzing mass amounts of data with strong clinical relevance, people can construct models that more accurately reflect tumor development and progression, thereby providing the possibility of personalized and sequential treatment of patients. As one of the cancer types whose treatment and diagnosis rely on imaging examination, radiomics has a very broad application prospect in head and neck cancers (HNC). Until now, there have been some notable results in HNC. In this review, we will introduce the concepts and workflow of radiomics and machine learning and their current applications in head and neck cancers, as well as the directions and applications of artificial intelligence in the treatment and diagnosis of HNC.

## Introduction

Head and neck cancer (HNC) is the eighth leading cause of cancer-related deaths 1. Now the main treatment modalities for head and neck tumors include surgery, radiotherapy, chemotherapy and immunotherapy 2. Due to tumor heterogeneity which serve as a known prognostic factor in HNC, a uniform treatment plan is not conducive to improved patient outcomes 3. Therefore, personalized treatment plan should be implemented for each patient to improve the survival time and minimize the side effects 4. In addition to tissue and blood tests, the diagnosis and treatment plan of HNC are also highly dependent on imaging, including computed tomography (CT), magnetic resonance imaging (MRI), and positron emission tomography (PET) 5. However, structural based medical images are traditionally evaluated subjectively and qualitatively, and in most cases, the experience of reader can greatly influence the results. In recent years, the emergence of radiomics has attracted attention, which can extract quantitative imaging features from conventional medical images, and these features can also be combined with pathology and molecular biomarker, so as to more accurately assess the biological state of tumors and make personalized diagnosis and treatment plans for patients 6-10.

In recent decades, radiomics has become a new and evolving field in medical imaging 11-12. With radiomics, people have discovered new image biomarkers, by collecting high-throughput quantitative features of oncology medical images. More and more research has shown that medical images contain more information than is available to the naked eye, and that extractable image parameters will in turn have some correlation with tumor clinical characteristics 11,13-15. Radiomics is designed to be used as a clinical decision support tool by extracting quantitative data from medical images. Mass data will also be generated in this process 7,11,16-17. Machine learning has promising application in radiomics due to its algorithms that are best suited for analysis of high-dimensional data 18-20. In recent years, due to the unprecedented development of machine learning algorithms, coupled with the fact that the data required already exist and are easily available, there have been many studies using radiomics in the diagnosis, treatment and prognosis of HNC. In this review, we summarized the radiomics research of HNC, and introduced its general principles and typical workflow, as well as its future prospects and limitations in the field of HNC.

## Workflow of radiomics and machine learning

Over the last decade, various “-omics” concepts have emerged one by one with the progress of high-throughput computer algorithms, which referring to the collective characterization and quantification of pools of biology information (e.g. proteomics, genomics, metabolomics). In recent years, more and more attention has been paid to radiomics, which refers to the automated extraction of mathematically defined radiological features from two- or three-dimensional (2D or 3D) medical images, as well as the application of data mining and analysis techniques 21-22. Radiomics consists of extracting hundreds of quantitative features through automated or semi-automated software. It based on a hypothesis is that mineable data can be extracted from medical images and provides additional information about tumors' phenotype, genes, and proteins for use in patients 15, 23-24. In recent years, more and more researchers have begun to focus on predicting molecular biomarkers, predicting therapeutic responses, and predicting survival prognostics in patients with HNC by extracting radiomics information features (including shape description, intensity, and texture characteristics) from different imaging patterns (e.g. CT, MRI, PET, ultrasound images) 11,13. Some subfields of radiomics focus on the identification and scientific exploitation of relationships between quantitative bioimaging and genomic features of tumors. Previous studies have shown that the characteristics of medical images can distinguish between the biological characteristics of some tissues, such as tumors, inflammation and necrosis. Sometimes these characteristics also could be used to study the correlation with disease diagnosis and prognosis 25-28. At the same time, some characteristics of medical images can be reflective of molecular and genetic characteristics of tumors 29-32.

In a typical radiomics workflow, image acquisition is often the first step and a critical one 33-34. Researchers must obtain high-quality, standardized imaging. The data source of radiomics is always obtained from retrospective medical imaging images. Different imaging techniques can lead to differences in image signals and image textures in medical imaging due to different acquisition parameters and reconstruction schemes 35-36. If the parameters collected vary widely, this can introduce signal changes that are not caused by biological effects. For radiomics image analysis, a large number of images need to be selected. These are ideally standardized for image characteristics in resolution, reconstruction and acquisition parameters, as well as clinical characteristics such as tumor stage, tumor classification or prognosis 37-39.

The next step is to delineate (“segmentation”) the target area and volume in a medical image, generating sub-parts of the image in 2D and 3D images called areas of interest (ROI) and volume of interest (VOI) 33-34. Segmentation must be reproducible and reliable, and it can be divided into manual, semi-automatic or automated execution. Manual separation requires two independent physicians (clinicians or imaging physicians) to complete, which can be time-consuming and labor-intensive, and the results are subject to observer variability and are not suitable for large-scale cohort studies. Semi-automatic image separation still requires human-machine coordination and an experienced physician is required to have an identification and modification of the automatically separated boundaries. Automatic image separation does not require human involvement, avoids heterogeneity between and within evaluators, and results are more repeatable, faster, and more suitable for large imaging datasets 34,40. The raw data needs to be preprocessed to distinguish the signals from the noise, and the selection of this step is very important because it will directly affect the extracted features 41.

Then the extraction of radiomics features would be implemented, which are usually performed fully automated by professional software 42-43. The radiomics features include shape features, which are used to represent the shape and geometry of ROI, such as head and neck tumor volume, length axis ratio, surface area/volume ratio, etc. 44. The first-order feature is used to study the distribution of voxel values without considering spatial relationships, such as the mean, median, standard deviation, and peak of the voxels strength.

**Figure 1:**
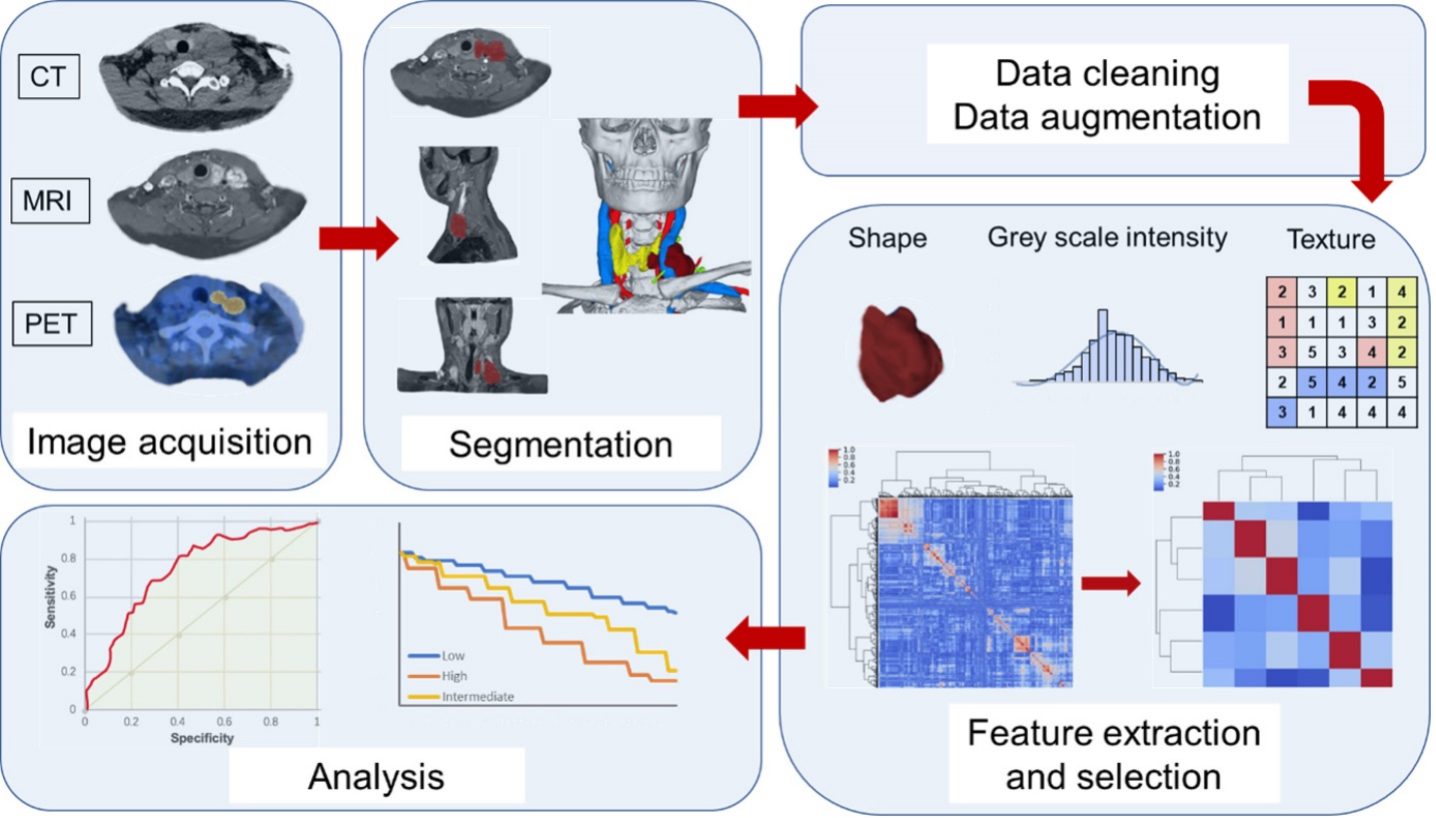
** Typical radiomics workflow.** ROI is first delineated. Then extract the features from the ROI, and finally model and analyzed.

Second-order features, or texture features, are used to analyze the characteristics of the spatial distribution relationship of voxel intensity between voxels, and can be used to measure heterogeneity within tumors, such as a co-occurrence matrix (GLCM) that could calculates the correlation between two gray levels at a certain distance and a certain direction in an image, calculates the gray-level run length matrix (GLRLM) of continuous voxels with the same intensity in a fixed direction, and the neighborhood gray-level different matrix (NGLDM) between the quantized voxel intensity and the average speed-up intensity of neighboring voxel within a certain distance 45-47. Deep learning is a sub-field of machine learning that has risen to the forefront of artificial intelligence, and one of the most popular deep learning tools available today, the convolutional neural network (CNN), can also be used to extract depth characteristics 48-49. Convolutional analysis is performed on the image through the CNN, and the data in the fully connected layer is used as the obtained depth feature. These features can continue to be used in the CNN or in other classifiers 50-51. In the stage of radiomics feature extraction, a large amount of data will be obtained. Before using these features, redundant features, unrelated or useless features should be excluded, leaving only a subset of features that are valid for modeling 42,44. In **Figure 1**, we summarized the general workflow of radiomics.

Radiomics extracts valid, quantitative features from medical images that can be combined with other routine prognostic markers such as clinical staging, tissue molecular markers, and pathological features 10. Various studies have shown that this type of predictive model based on medical images combined with various other data is superior in the evaluation of disease and survival prediction 13,53-55. It has been shown that machine learning is a powerful statistical tool that is required to effectively develop and apply such large amounts of high-dimensional data. The choice of modeling method depends on the type of data and the purpose of the study. Machine learning methods include decision trees (DT), random forests (RF), logistic regression, bayesian models, support vector machines (SVM) and recently, deep learning which has gained much attention 56-59. The technique has been widely used in the development of various predictive models for HNC.

## Application of radiomics and machine learning in head and neck cancers

Multiple radiomics studies in HNC have reported in various magazines recent years. These studies generally focus on the diagnostic prediction of radiomics in HNC (pre-treatment staging, pathological subtypes, differentiation of tumors from inflammation or necrosis), and prediction of tumor status after treatments (include the status of certain pathogenic viruses, the prediction of early recurrence or lymph node metastasis), the prediction of survival and adverse reactions after treatments. Some researchers have studied radiogenomics to explore the prediction of expression of some molecules in HNC.

## Pre-treatment related predictive modeling

Pre-treatment staging is an important part of tumor diagnosis and a factor closely related to tumor prognosis. Studies have shown that T-stage of head and neck tumors, viral-related status, and lymph node status greatly influence the prognosis of cancer patients 60-63. However, the current diagnostic methods focus on pre-treatment tissue biopsy, serological testing and traditional medical image diagnosis, which can determine tumor staging to a certain extent, but ultimately are local, qualitative and subjective. Reliable assessment of tumor staging by radiomics prior to treatment can help guide treatment selection and reduce recurrence and adverse event rates. Wang et al. 64 reported the use of radiomics combined with machine learning to create a T-staging model of locally advanced laryngeal cancer (LC), the performance of the model was evaluated by the area under the receiver operating characteristic curve (AUC). The predictive performance of the nomogram incorporating radiomic signature and T category reported by radiologists is the best with an AUC of 0.892 (95% CI: 0.811 to 0.974). Ren et al. 65 extracted imaging features from MRI of 85 patients in the training cohort and demonstrated that MRI radiomics signature could distinguish stage III- IV from stage I-II head and neck squamous cell carcinoma (HNSCC). Radiomics signature may serve as a complementary tool for preoperative staging. Romeo et al. 66 prediction of tumor grade and nodal status in oropharyngeal and oral cavity squamous-cell carcinoma using a radiomic approach. It has been reported that apparent diffusion coefficient based radiomics can be a useful and promising non-invasive method for predicting histologic grade of squamous cell carcinoma (SCC) of the oral tongue and tongue and floor of mouth. In HNSCC, radiological analysis was also used to design non-invasive biomarkers and to accurately distinguish well-differentiated from moderately differentiated and poorly differentiated HNSCC, with an AUC of 0.96 and an accuracy of 0.92. It has been reported that radiomics CT models have the potential to predict characteristics typically identified on pathologic assessment of HNSCC 67. In a cohort of 96 papillary thyroid carcinoma (PTC) patients, a prospective study enrolled consecutive patients who underwent neck MR scans and subsequent thyroidectomy during the study interval. Machine learning-based MRI prediction models can distinguish between aggressive and non-aggressive PTC before surgery, and this approach facilitates the formation of personalized PTC treatment plans 68. We identified eight studies investigated the feasibility of radiomics for the classification of HNC before treatment (**Table 1**). Thus far, these exploratory studies show that radiomics prediction model has the potential to become another non-invasive diagnostic tool for HNC before treatment, which can make the staging of tumors more objective and accurate, and even predict the malignancy of tumors and have a certain guiding effect on the subsequent treatment.

In the last 3 years, there have been an increasing number of studies to predict tumor response to certain treatments. It is well known that the treatment of HNC is mainly surgery, but there are also various treatment options with induction chemotherapy, concurrent chemoradiation, targeted therapy or immunotherapy 72-73. In order to better formulate personalized treatment plans for cancer patients, a sequential treatment system can be realized as soon as possible. The establishment of a radiomics model that can predict the treatment effect or the incidence of complication after treatment plays a very important role in achieving the above goals. While Bologna et al. Wang et al. and Zhao et al. 74-76 they retrospectively extracted radiomics signatures of each type of weighted images from MRI of nasopharyngeal carcinoma (NPC) patients. Selected the useful radiomics features by least absolute shrinkage and selection operator (LASSO) to form a valid model to predict early response of NPC patients to induction chemotherapy, which helps to personalize risk stratification and treatment of NPC patients. Jin et al. 77 reported their preliminary results based on radiomics features from CT scans in 70 patients with esophageal cancer (EC), they found that the model with radiomic features combined with dosimetric parameters is promising and outperforms that with radiomic features alone in predicting the treatment response of patients with EC who underwent concurrent chemoradiation. Acute xerostomia is the most common side effect of radiation therapy for HNC. Pota et al and Liu et al. 78-79 conducted a study on HNC and NPC, respectively. They obtained CT scans of patients before, during, and after treatment to obtain imaging features and establish the best model to use during the initial treatment phase to predict the development of acute xerostomia after radiation therapy in cancer patients. Radiation induced brain injury is a relatively common brain complication in patients with NPC after radiation therapy. Although this adverse reaction is not fatal in general, but it seriously affects patients' life treatment. Researchers extracted 10320 textural features by analyzing MRI multiple-weighted images of 242 patients with NPC who had undergone radiation therapy. Three prediction models were established using the RF method, all of which could dynamically predict radiation induced brain injury in advance, enabling early detection and allowing clinicians to take preventive measures to stop or slow down the deterioration of radiation induced brain injury 80. Eight studies explored the ability of radiomics to predict the response of HNC to certain treatments or early prediction of complication after treatment (**Table 2**).

## Prediction models for recurrence, metastasis and survival

The most extensive research of radiomics in HNC is its prediction of prognosis, including the study of its relationship with prognostic indicators such as progression-free survival (PFS), overall survival (OS), five-year survival rate, distant metastasis (DM), and local recurrence (LR). With the development of medical diagnosis and treatment technology, HNC has made great progress in the use of therapeutic drugs 82-84. However, due to the specificity of the growth site of HNC, many patients are already in advanced stage when they are found, which makes the prognosis of HNC still poor, with the five-year survival rate ranging from 25% for hypopharyngeal cancer to 80% for NPC 85-86. Therefore, scientists are interested in more accurately predicting LR, lymph node metastasis (LNM) and even distant metastasis (DM) of HNC and the survival rate of patients, which can also better serve in the development of personalized treatment plans for patients.

People used MRI images from 360 patients with NPC as a training cohort for feature extraction from the maximal axis region of the tumor. Eleven features were selected to construct the radiomics score (Rad-score), which was significantly associated with local recurrence-free survival (LRFS). Rad-scores were generated using the Cox proportional hazards regression model, and can reliably predict LRFS in patients with non-metastatic T4 NPC, which might guide individual treatment decisions 87. There are still a lot of imaging genomics combined with machine learning of various algorithms to build predictive models for LR of HNC. From the M.D. Anderson cancer center head and neck quantitative imaging working group, which analyzed CT/MRI and PET images from 465 patients with HNC. Machine learning methods were applied to yield a radiomic signature consisting of features with minimal overlap and maximum prognostic significance, and derived from pre-treatment imaging consisting of 2 radiographic signatures 88. In thyroid and EC, predictive models of radiomics have also been used 89-94. LNM is a significant prognostic factor in patients with HNC, and the ability to predict it accurately is essential to optimizing treatment. The accuracy of LNM identification strongly depends on the physician's experience, therefore, the establishment of an automatic prediction model for LNM can greatly help physicians in their practice 34. Radiomics models are built based on handcrafted features, while deep learning learns the features automatically. In order to have better prediction, many researchers have proposed hybrid prediction models 95-98. Another research group used the PyRadiomics platform, and extracted the imaging features of primary tumors in all patients who did not exhibit DM before treatment. This retrospective cohort analysis included 176 patients with NPC. Then used minimum redundancy-maximum relevance and LASSO algorithms to select the strongest features and build a logistic model for DM prediction 99. From these existing exploratory studies, it is easy to see that most researchers have extracted radiomics signatures manually or semi-manually from various types of imaging images of training cohorts, then used machine learning algorithms to extract valid features and build predictive models, and then used independent cohorts to verify the validity of the models. **Table 3** summarizes the reported studies of representative predictive models of this type in HNC.

In research reports on the use of radiomics in HNC, radiomics models related to predicting survival are the most numerous. Shen et al. 108 aimed to explore the predictive value of MRI-based radiomic model for PFS in nonmetastatic NPC. They collected the clinical and MRI data from 327 patients with NPC, and five models were established. The prognostic performances of these models were evaluated by Harrell's concordance index (C-index). They find that the model incorporating radiomics, overall stage, and EBV DNA showed better performance for predicting PFS in nonmetastatic NPC patients. In HNSCC, Yuan et al. 109 consisted of a training cohort (n = 85), and LASSO Cox regression model was used to select the most useful prognostic features with their coefficients, upon which a radiomic signature was generated. They find that MRI-based radiomic signature is an independent prognostic factor for HNSCC patients. Another study identified prognostic and reliable machine-learning methods for the prediction of overall survival of head and neck cancer patients 110. Others have used pre- and post-operative PET/CT radiomics features for HNSCC and found that combining clinicopathological characteristics with radiomics features of pre-treatment PET/CT or post-treatment PET/CT assessment of primary tumor sites as positive or negative may substantially improve prediction of OS and DFS of HNSCC patients 111-112. The predictions of radiomics signature models based on various types of imaging sequences in various types of HNC are represented in **Table 4.** The main types of survival values predicted by each type of model and which machine learning algorithms were employed are specified in the table.

## Other predictive models

Tumor heterogeneity is a well-known prognostic factor in HNC. A major limitation of tissue- and blood-derived tumor markers is the lack of spatial resolution to image tumor heterogeneity. Due to the hidden growth sites of HNC, it is difficult to obtain biopsies before and after treatment. At the same time, issue markers derived from tumor biopsies usually represent only a small tumor subregion at a single timepoint and are therefore often not representative of the tumors' biology or the biological alterations during and after treatment. This has also been noted by researchers, Gu et al. 138 showed that a radiomics model with excellent performance prediction of the presence of cytokeratin 19, galectin 3, and thyro-peroxidase based on CT images. This model may be used to identify benign and malignant thyroid nodules. Chen et al. 31 investigated the correlation between programmed cell death protein 1 ligand (PD-L1) immunohistochemical expression and PET/CT radiomics and found that p16 and Ki-67 staining percentages and several PET/CT-derived textural features could provide additional information to identify tumor PD-L1 expression in HNC. There are also several researchers have done studies correlating radiomics features with molecular features of HNC 10,32,139. In recent years, some researchers have begun to focus on the comparison of the predictive performance of radiomics models of different image modalities in the same disease 36, 53,140.

## Discussion

In recent years, numerous literatures revealed that radiomics has been studied in the pre-treatment diagnosis of head and neck cancer, including the prediction of efficacy and the prediction of survival. These studies have yielded promising results and have drawn good lessons for subsequent researchers. However, there is still a lack of large-scale multicenter validation in existing exploratory radiomics studies, and the vast majority of validation cohorts are still derived from retrospective data from a single independent unit. A data platform such as the cancer imaging archive (TCIA) has been created, but the quality of the data profile is mixed 67. Although relatively reliable conclusions can be drawn from some of the mixed data by relying on big data techniques, however, differences in parameters during image acquisition or noise on the images can cause serious interference with the radiomics features extracted from them. This interference will inevitably affect the model's ability to generalize to other databases as well.

The relationship between radiomics and clinical symptoms has been widely documented, but other data types, such as genomics, transcriptomics, proteomics, and metabolomics, have been less studied in relation to radiomics. In HNC, correlation studies between imaging and genomics are now available, as important molecular markers such as PD-L1/TP53/FAT1/KMT2D/NOTCH1/Ki-67 can be predicted by predictive models of imaging features 10,32,141. At present, the relationship between imaging and transcriptomics has been studied in other tumors, but its combination with proteomics and metabolomics is still less studied. This may be related to the fact that currently the histological data are independent of each other, and samples with these histological data do not have radiomics data.

The next milestone in radiomics is undoubtedly the creation of decision support and predictive tool models. In order to achieve this goal, having big data of all types of data is a sine qua non, and a strong and comprehensive common database is an effective solution. To achieve this goal, in addition to the involvement of different medical centers from all over the world to provide data, a worldwide accepted standard should be developed first. This standard should establish more uniform regulations in radiomics from the acquisition of source data, segregation of regions of interest, extraction of features to the development of predictive models. Although the difficulty and cost of creating and managing high-quality public data is enormous, the benefits to human medicine are also enormous.

People have investigated the association between PD-L1 expression in HNC patients and PET/CT, but did not delve into the efficacy of immunotherapy 31. In other tumors, such as glioma and non-small-cell lung cancer, the models found in these studies have potential important translational implications to identify highly vulnerable patients treated with immunotherapy that experience rapid disease progression and survival poor outcomes 142-143. These studies demonstrated that clinical data combined with radiomics performed better than traditional clinical data in predicting the efficacy of immunotherapy. Immunotherapy, as a new therapy in modern cancer treatment, has been shown to be less effective in many solid tumors, such as HNC, and therefore the development of appropriate models to predict the efficacy of immunotherapy prior to treatment would be of great help in avoiding the waste of medical resources and developing more accurate and personalized treatment plans.

In the current study of radiomics in HNC, it can be found that the vast majority of studies are still based on a single imaging modality, with few studies combining multiple imaging modality characterization. The predictive power of multiple imaging modalities in the same disease is still unknown, and our current research direction is trying to fill this vacancy. At the same time, as mentioned above, the development of predictive models by combining imaging modalities with multi-modality studies is still in its infancy, and there is still a lot of room for improvement, which is the direction we are working on at our medical center. The easier access to the data required for radiomics, unlike routine biopsies or other histology, provides new directions for otolaryngologists and craniofacial surgeons to study underlying tumors of the skull (in addition to routine HNC). As we all know, compared with HNC, skull base tumors are more difficult to biopsy and diagnose, and because of their insidious development, patients often do not show symptoms until later stages. In addition, the special and complex anatomical structure of the skull base often makes it more difficult for skull base surgeons to estimate the nature of the tumor and determine the scope of resection before surgery. Although relevant studies have been done by researchers, such as Li et al. 11 selected features were finally selected from skull base MRI of 210 patients to establish a radiomics model to differentiate between skull base chordoma and chondrosarcoma 144. Other researchers have used MRI radiomics to predict the likelihood of early progression or recurrence in a subset of patients with skull base meningiomas due to incomplete resection 145-146. However, the application of radiomics in skull base tumors is still rare, which may be due to the special location of skull base tumors, and the image range including various neurovascular, brain tissue, bone and even nasal and orbital conditions, this results in a complex image texture. Because of these complexities, it is necessary to develop radiomics, which can be used to obtain objective information through non-invasive testing, combined with machine learning to build pathological classification prediction models or conventional prognostic models, to guide the selection of treatment, design the scope of surgery, and even guide the postoperative comprehensive treatment. This is also very much in line with the concept of sequential cancer treatment.

With the enhancement of radiomics technology, the expansion of public databases, and the advancement of deep learning algorithms, radiomics will certainly play an important role in the future clinical diagnosis, treatment and prognosis. Radiomics is expected to lay the foundation for the future personalized treatment of otolaryngology patients and the sequential treatment of tumors.

## Figures and Tables

**Table 1:** Radiomics assesses pre-treatment grading of head and neck cancers

Study	Number of patients	Tumor characteristics	Imaging modality	Parameter prediction	Feature selection method, model	Machine learning algorithm
Wang et al. 2019 64	Train:150pts Validation:61pts	Locally advanced LC	CE-CT	T stage	LASSO. Multivariable logistics model	LASSO, SVM, CHAID
Ren et al. 2018 65	Train: 85pts Validation: 42pts	HNSCC	T2, CE- T1	T stage	LASSO. Rad-score	LASSO
Wu et al. 2019 69	Train: 137pts Validation: 69pts	HNSCC	CE-CT	Degree of tumor differentiation	KPCA, random forest classifier and VT. Multivariable logistics model	RF, K-PCA
Mukberjee et al. 2020 67	Train: 113pts Validation: 71pts	HNSCC	CE- CT	Histopathologic features	PCA. Regularized regression	PCA
Katsoulakis et al. 2020 71	Train: 77pts Validation: 83pts	HNSCC	CE- CT from TCIA	Molecular differences	CERR. Logistic regression	RF, deconvolution analysis, logistic regression
Ren et al. 2020 70	Train: 59pts Validation: 29pts	SCC of the oral tongue and floor of mouth	T1, T2, CE- T1, DWI	Histologic grade	LASSO. Rad-score	LASSO
Romeo et al. 2020 66	Total: 40pts	Oropharyngeal and oral cavity SCC	CE- CT	T stage and Nodal status	Heterogeneity CAD. Machine learning classifiers	Naïve bayes, KNN, RF and so on
Wang et al. 2019 68	Train: 96pts Validation: 24pts	PTC (prospective)	T2, CE-T1, DWI	Aggressiveness level	LASSO and selection operator. Machine learning classifiers	LASSO, gradient boosting classifier, logistic regression

Train: Training dataset; Total: Only one dataset used; Validation: Validation dataset; CE-CT: Contrast-enhanced CT; CE-T1: Contrast-enhanced T1; DWI: Diffusion weighted imaging; HPV: Human Papillomavirus; PCA: Principal component analysis; CERR: Computational environment for radiological research; CAD: Computer-aided diagnosis and detection systems; CHAID: Chi-square automatic interaction detection; KNN: K-nearest neighbor.

**Table 2:** Radiomics predicts tumor response and adverse symptom after treatment

Study	Number of patients	Tumor characteristics	Imaging modality	Therapy/Symptom	Outcome, model	Machine learning algorithm
Yu et al. 2019 81	Train: 51pts; Validation: 19pts	NPC	T2, CE-T1	ART	Replan status of patient. Multivariable logistics model	LASSO, logistic regression
Zhang et al. 2020 80	Total: 242pts	NPC	T2, CE-T1	Early detection of radiation- induced brain injury	Radiation induced temporal lobe injury. Random forest	RF
Pota et al. 2017 78	Total: 37pts (74 parotid glands)	HNC	Before RT (CT1), at the middle of treatment (CT2) and after RT (CT3)	Xerostomia, shrinkage of parotid glands	Parotid shrinkage rate and 12-months xerostomia. Machine learning classifiers	Naïve bayes, LFA
Jin et al. 2019 77	Train: 70pts Validation: 24pts	EC	Before treatment (CT1), 3 months after CRT(CT2)	Concurrent chemoradiation	3 months after CRT, Machine learning classifiers	SVM, RBF, XGBoost, PCA
Liu et al. 2019 79	Total: 35pts	NPC	Five CT sets acquired at treatment position during the RT	Acute xerostomia	Patients' saliva was collected every other 10 days during the RT. Multivariate Cox regression	Multivariate machine learning algorithms
Bologna et al. 2020 74	Total: 50pts (25 responders and 25 non-responders)	Sinonasal cancers	T1, T2, ADC	Induction chemotherapy	T1+T2+ADC model displayed the highest; radiomics score	PCA, naïve bayes, SVM, KNN and so on
Wang et al. 2018 75	Total: 120pts	NPC	T2, T2FS, CE- T1	Induction chemotherapy	Early response to induction chemotherapy; logistic regression	Logistic regression
Zhao et al. 2019 76	Train: 100pts Validation: 23pts	NPC	T1, T2, CE- T1	Induction chemotherapy	PFS; multivariable logistic regression	LASSO, SVM, logistic regression

ART: Adaptive radiotherapy; LFA: Likelihood-fuzzy analysis; RBF: Radial basis function; XGBoost: Extreme gradient boosting algorithm; CRT: Concurrent chemoradiation; T2FS: T2 weighted fat-suppressed; RT: Radiation therapy; EBV: Epstein-Barr virus; ADC: Apparent diffusion coefficient.

**Table 3:** Radiomics predicts recurrence and metastasis of head and neck cancer

Study	Number of patients	Tumor type	Imaging modality	Outcome, feature selection method, model	Machine learning algorithm
Zhang et al. 2019 87	Train: 80pts Validation: 60pts	NPC	T2, CE- T1	LR-free survival. Radiomics score, Cox regression	Logistic Regression
Bogowicz et al. 2017 100	Train: 93pts Validation: 56pts	HNSCC	CE- CT	LC and HPV status. PCA in combination with univariable logistic regression. Multivariable logistic regression	Logistic regression, PCA
Martens et al. 2020 101	Train: 103pts Validation: 71pts	HNSCC	PET, low-dose-CT	LR, DM, OS. RadCat tool Cox regression analysis. Multivariable logistic regression	Logistic regression
Li et al. 2018 97	Total: 306pts, 20 of whom developed with recurrence	NPC	CT, MR, PET	LR. PCA. Machine learning classifiers	PCA, ANN, KNN, SVM
Liu et al. 2019 90	Total: 120pts	PTC	Preoperative ultrasound images	Metastasis. Support vector machine classifier	SVM
Wu et al. 2020 98	Train: 141pts Validation: 96pts	HNC	PET, CT	LR. PCA. Multivariate Cox proportional hazards regression	PCA
Zhou et al. 2020 95	Total: 188pts	HNSCC	PET, CT	DM. Machine learning classifiers	SVM, DT and KNN
Bogowicz et al. 2017 102	Train: 128pts Validation: 50pts	HNSCC	PET, CT	LR. PCA and LASSO. Multivariable Cox regression	PCA, LASSO
Tan et al. 2018 89	Train: 154pts Validation: 76pts	ESCC	Arterial-phase CT	LMR. Rad-score, logistic regression	LASSO, logistic regression
Vallieres et al. 2017 103	Total: 300pts	HNC	Pre-treatment FDG-PET and CT	LR and DM. Machine learning classifier	Random forests
Kwan et al. 2018 104	Total: 300pts 36 DM pts	HPV-related Oropharyngeal Carcinoma	CT	DM. PyRadiomic. Radiomics score	Logistic regression
Park et al. 2019 92	Train: 400pts Validation: 368pts	PTC	Neck ultrasound	LNM. LASSO. Rad-score, LASSO regression	LASSO
Zhang et al. 2019 105	Train: 360pts Validation: 120pts	Non-metastatic T4 NPC	T1, T2, CE- T1	LR, Rad-score, cox proportional hazards regression	Logistic regression
Zhang et al. 2019 99	Total: 176pts	NPC	PET, CT	DM. LASSO. Multivariate logistic regression	Logistic regression
M.D. Anderson Cancer 88	Train: 255pts Tune: 165pts Validation: 45pts	HNC	CT, MRI, PET	5-year LCR. Multivariable Cox regression	DT
Bahig et al. 2019 106	Total: 176pts, 20 supraglottic , 5 pyriform sinus tumors	LHSCC	DECT	LRR. Univariate Cox regression	DT
Lu et al. 2019 93	Train: 154pts Validation: 67pts	PTC	non-contrast and venous CE- CT	LNM. SVM. Multivariable logistic regression	SVM, logistic regression
Qu et al. 2018 94	Train: 90pts Validation: 91pts	EC	MRI	LNM. LASSO. Multivariable logistic regression	LASSO, elastic net regression, logistic regression
Martens et al. 2020 107	Train: 103pts Validation: 71pts	HNSCC	18F-FDG-PET, CT	LR, DM, OS. Rad- score. Multivariable survival regression	Logistic regression

ANN: Artificial neural network; LCR: Local control rate; DECT: Dual-energy computed tomography; LHSCC: Larynx and hypopharynx squamous cell carcinoma.

**Table 4:** Radiomics predicts the survival of head and neck cancer

Study	Tumor characteristics	Imaging modality	Outcome	Machine learning methods
Shen et al. 2020 108	NPC	MRI	PFS	LASSO
Xu et al. 2020 113	NPC	PET	PFS	/
Ouyang et al. 2017 114	NPC	MRI	PFS	/
Zhang et al. 2017 115	Advanced NPC	MRI	PFS	LASSO
Lv et al. 2019 53	NPC	PET	PFS	/
Peng et al. 2019 52	NPC	PET	DFS	Deep learning
Yuan et al. 2019 109	HNSCC	MRI	OS	LASSO
Ming et al. 2019 116	NPC	MRI	DFS, OS, LRFS, DMFS	LASSO
Mao et al. 2019 117	NPC	MRI	PFS	/
Chen et al. 2020 118	LC	CT	OS	LASSO
Folkert et al.2017 119	OC	PET	ACM, DM	Multiparameter logistic regression
Foley et al.2018 120	OC	PET	OS	/
Chen et al.2019 121	EC	PET	DFS, OS	Multivariate logistic regression
Xiong et al. 2017 122	EC	PET	PFS	SVM, RF
Feliciani et al. 2018 123	HNC	PET	PFS	LASSO
Liao et al. 2019 124	Oropharyngeal and hypopharyngeal cancer	PET	OS, PRFS, DFS	/
Lv et al. 2019 125	HNC	PET/CT	RFS, MFS, OS	/
Yang et al. 2019 126	Advanced NPC	MRI	PFS	LASSO
Leijenaar et al. 2015 127	OSCC	CT	OS	/
Agarwal et al. 2020 111	LC	CT	LFS	/
Zhong et al. 2020 128	T3N0M0 NPC	MRI	DFS	Deep learning
Parmar et al. 2015 110	HNC	CT	OS	Different machine-learning classifiers
Liu et al. 2020 112	HNSCC	PET	OS, DFS	/
Pan et al. 2019 130	Oral tongue cancer	CT	Survival time	PCA
Xie et al. 2020 129	HNC	PET	OS, DFS	LR, SVM, RF, XG boost classifier
Cozzi et al. 2019 131	HNC	CT	OS, PFS	/
Legar et al. 2018 132	HNC	CT	OS, LRC	Six machine learning algorithms
Sörensen et al. 2019 133	HNC	PET	OS	/
Haider et al. 2020 134	OSCC	PET	OS, PFS	/
Ou et al. 2017 135	HNC	CT	PFS, OS	PCA
Miller et al. 2019 136	OPSCC	CT	PFS	/
Mes et al. 2020 137	HNSCC	MRI	RFS, OS	/

OPSCC: Oropharyngeal Squamous Cell Carcinoma; RFS: Relapse-free survival; ACM: All-cause mortality; LFS: Laryngectomy free survival; PRFS: Primary relapse-free survival; RFS: Recurrence-free survival; MFS: Metastasis-free survival; LRC: Locoregional tumor control; DMFS: Disease distant metastasis-free survival; OC: Oropharyngeal carcinoma.

## Abbreviations

HNC: head and neck cancers
CT: computed tomography
MRI: magnetic resonance imaging
PET: positron emission tomography
2D or 3D: two- or three-dimensional
ROI: areas of interest
VOI: volume of interest
GLCM: co-occurrence matrix
GLRLM: gray-level run length matrix
NGLDM: neighborhood gray-level different matrix
CNN: convolutional neural network
DT: decision tree
RF: random forests
SVM: support vector machines
LC: laryngeal cancer
AUC: area under the receiver operating characteristic curve
HNSCC: head and neck squamous cell carcinoma
SCC: squamous cell carcinoma
PTC: papillary thyroid carcinoma
NPC: nasopharyngeal carcinoma
PD-L1: programmed cell death protein 1 ligand
LASSO: least absolute shrinkage and selection operator
EC: esophageal cancer
PFS: progression-free survival
OS: overall survival
DM: distant metastasis
LR: local recurrence
LNM: lymph node metastasis
Rad-score: radiomics score
LRFS: local recurrence-free survival
DFS: disease-free survival
TCIA: The Cancer Imaging Archive
CE-CT: contrast- enhanced CT
CE-T1: contrast-enhanced T1
DWI: diffusion weighted imaging
HPV: human Papillomavirus
PCA: principal component analysis
VT: variance-threshold
CERR: computational environment for radiological research
CHAID: chi-square automatic interaction detection
KNN: k-nearest neighbor
CAD: computer-aided diagnosis
ART: adaptive radiotherapy
LFA: likelihood-fuzzy analysis
RBF: radial basis function
CRT: concurrent chemoradiation
T2FS: T2 weighted fat-suppressed
RT: radiation therapy
EBV: Epstein-Barr virus
ADC: apparent diffusion coefficient
ANN: artificial neural network
LCR: local control rate
DECT: dual-energy computed tomography
LHSCC: larynx and hypopharynx squamous cell carcinoma
OPSCC: Oropharyngeal Squamous Cell Carcinoma
RFS: relapse-free survival
ACM: all-cause mortality
PRFS: primary relapse-free survival
RFS: recurrence-free survival
MFS: metastasis-free survival
DMFS: disease distant metastasis-free survival
OC: oropharyngeal carcinoma
